# Efficacy of an enhanced linkage to HIV care intervention at improving linkage to HIV care and achieving viral suppression following home-based HIV testing in rural Uganda: study protocol for the Ekkubo/PATH cluster randomized controlled trial

**DOI:** 10.1186/s12879-017-2537-z

**Published:** 2017-07-03

**Authors:** Susan M. Kiene, Seth C. Kalichman, Katelyn M. Sileo, Nicolas A. Menzies, Rose Naigino, Chii-Dean Lin, Moses H. Bateganya, Haruna Lule, Rhoda K. Wanyenze

**Affiliations:** 1 Division of Epidemiology and Biostatistics, Graduate School of Public Health, San Diego State University, 5500 Campanile Drive (MC-4162), San Diego, CA 92182 USA; 2 Department of Psychology, University of Connecticut, Storrs, CT USA; 3 Division of Epidemiology and Biostatistics, Graduate School of Public Health, San Diego State University, San Diego, CA USA; 4 Department of Global Health and Population, Harvard T. H. Chan School of Public Health, Boston, MA USA; 5 Department of Disease Control and Environmental Health, Makerere University School of Public Health, Kampala, Uganda; 6 Department of Mathematics and Statistics, San Diego State University, San Diego, CA USA; 7 Formerly: Department of Global Health, University of Washington, Seattle, WA USA; 8 Gombe Hospital, Gombe, Uganda

**Keywords:** Home-based HIV counseling and testing (HBHCT), Linkage to care, HIV viral suppression, Health behavior intervention, Cluster-randomized controlled trial, Uganda

## Abstract

**Background:**

Though home-based human immunodeficiency virus (HIV) counseling and testing (HBHCT) is implemented in many sub-Saharan African countries as part of their HIV programs, linkage to HIV care remains a challenge. The purpose of this study is to test an intervention to enhance linkage to HIV care and improve HIV viral suppression among individuals testing HIV positive during HBHCT in rural Uganda.

**Methods:**

The PATH (Providing Access To HIV Care)/Ekkubo Study is a cluster-randomized controlled trial which compares the efficacy of an enhanced linkage to HIV care intervention vs. standard-of-care (paper-based referrals) at achieving individual and population-level HIV viral suppression, and intermediate outcomes of linkage to care, receipt of opportunistic infection prophylaxis, and antiretroviral therapy initiation following HBHCT. Approximately 600 men and women aged 18-59 who test HIV positive during district-wide HBHCT in rural Uganda will be enrolled in this study. Villages (clusters) are pair matched by population size and then randomly assigned to the intervention or standard-of-care arm. Study teams visit households and participants complete a baseline questionnaire, receive HIV counseling and testing, and have blood drawn for HIV viral load and CD4 testing. At baseline, standard-of-care arm participants receive referrals to HIV care including a paper-based referral and then receive their CD4 results via home visit 2 weeks later. Intervention arm participants receive an intervention counseling session at baseline, up to three follow-up counseling sessions at home, and a booster session at the HIV clinic if they present for care. These sessions each last approximately 30 min and consist of counseling to help clients: identify and reduce barriers to HIV care engagement, disclose their HIV status, identify a treatment supporter, and overcome HIV-related stigma through links to social support resources in the community. Participants in both arms complete interviewer-administered questionnaires at six and 12 months follow-up, HIV viral load and CD4 testing at 12 months follow-up, and allow access to their medical records.

**Discussion:**

The findings of this study can inform the integration of a potentially cost-effective approach to improving rates of linkage to care and HIV viral suppression in HBHCT. If effective, this intervention can improve treatment outcomes, reduce mortality, and through its effect on individual and population-level HIV viral load, and decrease HIV incidence.

**Trial registration:**

NCT02545673

## Background

In the fight against human immunodeficiency virus (HIV), efforts to expand access to antiretroviral treatment (ART) are being prioritized globally. ART has the ability not only to improve health outcomes for the individual, but also in the prevention of ongoing HIV transmission through reducing viral load (VL). The push towards universal access to ART is reflected in the United Nations Joint Programme on HIV/Acquired Immunodeficiency Syndrome (AIDS) “90-90-90” goals, which aim to get 90% of people living with HIV (PLHIV) aware of their status, 90% of those diagnosed linked to care and receiving HIV treatment, and 90% of those receiving treatment virally suppressed by 2020 [1]. The 2016 World Health Organization (WHO) consolidated guidelines recommend all PLHIV be started on ART immediately upon diagnosis, regardless of CD4 cell count or disease stage [2]. However, significant challenges in getting PLHIV linked to HIV care and on ART remain, including in sub-Saharan Africa (SSA), the region most affected by the HIV epidemic. It is estimated that only 53% of those eligible for ART in SSA were receiving it in 2015 [3]. Several systematic reviews of studies in SSA demonstrate low rates of linkage to care after HIV testing [4–7], with up to two-thirds of patients lost to follow up before initiation of ART [7]. Thus, strategies to facilitate linkage into HIV care in SSA are needed, especially as the number of people eligible for ART grows as countries implement “test and treat” recommendations throughout the region.

In Uganda, it is estimated that only 65% of the 1.46 million PLHIV were aware of their status in 2015, of which only 51% were receiving ART [8]. Moreover, the number of people eligible for ART is expected to increase, as the revised 2016 Ugandan Ministry of Health (MOH) national treatment guidelines, which take effect in 2017, promote expansion of ART to all PLHIV, and put forth a number of strategies to increase the number of people aware of their HIV status and on treatment [8]. Home-based HIV counseling and testing (HBHCT) is one such approach, where HIV counseling and testing teams travel door-to-door and offer HIV testing services. HBHCT is especially important in the era of expanded access to ART and “treatment as prevention,” since HBHCT, compared to other modalities of testing, identifies HIV infections at an earlier disease stage and reaches more people who have never had an HIV test [9]. Moreover, HBHCT may be the most cost-effective testing strategy per person tested [9] and per new HIV infection identified [10]. However, though HBHCT is being widely implemented throughout SSA, [11] very few studies have examined linkage to care and treatment for individuals who test HIV positive at home [12–14].

The most common linkage-to-care approach with HBHCT, paper-based referral to care sometimes with follow-up home visits, has not been rigorously evaluated, and a number of HBHCT-specific barriers may limit its effectiveness. A systematic review on linkage to care in developing countries identified the following as the most commonly cited barriers: psychosocial factors (fear of being seen at the clinic, fear of disclosing, not feeling sick), structural and economic barriers (transportation costs, distance to the clinic), and health system factors (drug stock-out) [5]. Research evidence in Uganda has similarly documented these barriers [15], with pervasive HIV stigma, especially in rural areas, a particularly prominent barrier to care [16–19]. Such barriers may limit implementation of HBHCT; while HBHCT minimizes stigma and structural barriers of HIV testing (e.g., transportation) by bringing the test directly to patients [20], such barriers may remain an issue for linkage to care, as testing occurs away from the health facility and in a community setting, where fear of stigma may be elevated. Moreover, there is less structure in place for tracking patients testing positive in HBHCT, which is often done once without any follow-up [21]. In Uganda, HBHCT has been shown to be highly acceptable; 94% of individuals offered testing in a district-wide HBHCT study accepted testing and received their results [22]. However, only 10.5% of those who tested HIV positive were linked to ART [22]. In another study, only 58% of those who tested positive in HBHCT were linked to care within 12 months [23]. In HBHCT studies across SSA, linkage ranges from 42% to 97% within 1-12 months follow-up [24–30].

Low rates of linkage to HIV care during HBHCT highlights the need for interventions aimed to improve access to HIV care and treatment for individuals testing HIV positive in the home. The literature on HBHCT programs suggests that those with two follow-up home visits with counseling have better linkage compared to those with one or no follow-up home visits [24–30]. This finding is supported by recent trials in South Africa and Uganda, which found lay counselor facilitation and follow-up visits increased linkage [14, 31]. Based on evidence of the strong influence of HIV stigma, linkage to care interventions should be optimized in the context of rural HBHCT if they are to effectively reduce HIV stigma while addressing other barriers to care. A review of interventions to reduce HIV stigma found those providing social support to PLHIV were effective at reducing stigma [32], and emotional support through support groups has been shown to reduce internalized stigma among HIV-positive women in South Africa [33]. While no intervention studies have further linked reductions in stigma to subsequent improvements in linkage to care, a randomized trial in Uganda showed that counseling and social support via home visits doubled the likelihood of linkage to care for newly-diagnosed PLHIV [34]. Other data similarly suggests that social support may increase care linkage and retention [35–37], as well as reciprocal relationships between social support and HIV stigma [38]. Thus, providing PLHIV social support soon after diagnosis may protect them from internalizing HIV stigma and buffer the effects of enacted HIV stigma, which in turn may improve linkage to HIV care.

This paper describes the protocol for a cluster-randomized controlled trial (CRCT) to test an enhanced linkage to care intervention during HBHCT in rural Uganda. Our Providing Access To HIV care (Providing Access to HIV Care (PATH)/ *Ekkub*o) intervention enhances a similar strategy tested by Coates, Wanyenze, Bangsberg, and colleagues [39] that was found to reduce time to HIV care and treatment among patients receiving opt-out HIV testing in health facilities in Uganda. The intervention facilitated linkage by: providing orientation to the HIV care system, counseling to help clients identify and reduce barriers to engagement in care, and assistance disclosing and identifying a treatment supporter. The PATH/Ekkubo intervention described in this paper enhances the original intervention in order to increase its impact on multiple outcomes, including HIV viral suppression, and adapts the intervention to address barriers specific to HBHCT by providing additional counseling with increased emphasis on HIV stigma reduction through increased social support. We test this intervention in a CRCT, with randomization at the level of the village rather than at individual level, to avoid potential contamination since village residents interact with one another regularly in our study setting. The specific aims are:

1. Compare the efficacy of the enhanced linkage to care intervention (PATH) vs. standard-of-care (paper-based referrals) at achieving individual and population-level HIV viral suppression, and intermediate outcomes of linkage/time to care, time to/receipt of ART. We hypothesize that the PATH intervention study arm will have a higher proportion of participants who are: a) virally suppressed at 12-month follow up, and will have shorter time to: b) HIV care and c) ART initiation, among those eligible, than the standard-of-care study arm. We also hypothesize that the effect of the intervention on HIV viral suppression will be mediated by intermediate behavioral outcomes: linkage to care and ART initiation. Finally, at the cluster level, we hypothesize that villages randomized to the intervention arm will have higher rates of population-level HIV viral suppression than those in the standard-of-care study arm at 12-month follow-up.
2. Using the standard-of-care group as a natural history control, collect longitudinal data on barriers to and facilitators of linkage to and retention in care and treatment and HIV viral suppression.
3. Estimate the cost and cost-effectiveness of the intervention, as compared to standard-of-care HBHCT, in terms of: viral suppression, linkage to care, and receipt of ART.

## Design and methods

### Setting

The study is being conducted in the rural districts of Butambala, Mpigi, Mityana, and Gomba in central Uganda. Health services, including HIV care and treatment, are provided free at government health facilities. In line with WHO recommendations [40], the Ugandan MOH 2016 guidelines, now expand access to lifelong ART to all adolescents and adults living with HIV immediately upon diagnosis regardless of CD4 count, in contrast to their previous eligibility threshold of CD4 < 500 [8]. Our study began before these new guidelines were nationally disseminated and implemented. Under the currently implemented MOH protocol, at the first clinic visit, a CD4 test is done and patients are started on co-trimoxazole. Once CD4 results are available (<2 weeks), those eligible for ART receive pre-treatment counseling and are given an appointment to come back 1 week later to initiate ART. HIV care and treatment, as well as peer support groups for PLHIV, are available at all district hospitals and level III health centers. As the 2016 policy for the expansion of lifelong ART is rolled out during 2017 all HIV-positive individuals will become eligible for ART regardless of their CD4 count. Throughout Uganda, a cadre of lay workers, constituting the Village Health Team (VHT), support community health-related activities and serve as liaisons between the community and the health facilities.

### Study design

**Fig. 1:**
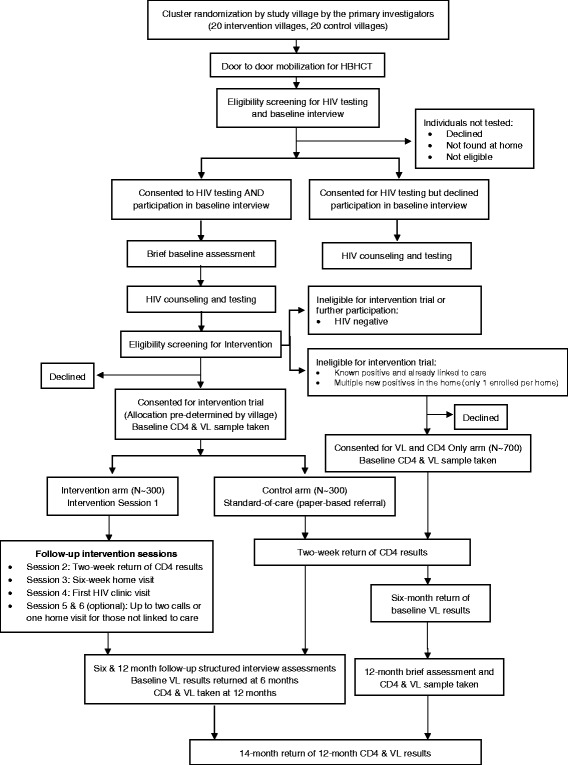
Consort Diagram of the Study Design

The trial design is a matched-pair two-arm, cluster-randomized design with clusters being villages. One arm is the PATH/Ekkubo intervention arm and the other is a standard-of-care (control) arm. Villages are randomized to study arms. We will include 40 villages with approximately 600 participants across all clusters (villages). We will conduct structured interviews at baseline (pre-intervention), and at six and 12-month follow-up. Similarly, blood draws for HIV viral load and CD4 testing will be done at baseline and at 12-month follow-up. The design is illustrated in the CONSORT Diagram, Fig. 1.

### Study population

The study population is adults aged 18 to 59 as well as emancipated minors (which according to the Ugandan National Council for Science and Technology are those under 18 who are married, have children, or are pregnant), residing in villages selected for inclusion in the study. Individuals who have accepted HBHCT and provide written (or thumb-printed) informed consent will be eligible to participate in the study.

### Sampling and randomization

The sampling scheme is stratified clustering with matched-pair sampling. Within each district we will pair match the 40 clusters (villages) by population size and proximity to major and regional roads, since the latter may facilitate access to health services because of greater availability of transportation. Using computer generated random numbers, we will randomly assign one village to be in the intervention arm and the other to the standard-of-care arm. Since village residents interact with one another regularly, we chose to randomize at the level of the village instead of randomizing individuals to study condition to avoid potential contamination. We will ensure that villages assigned to different study conditions are not within 4 km (1 h. by foot) of each other.

### Procedures

#### Community mobilization for HBHCT

Following the Ugandan MOH guidelines [41], this study provides village-wide HBHCT across the study villages. HBHCT and study recruitment is being conducted over approximately 46 months, beginning in November 2015 and ongoing through August 2019. The HBHCT teams will work in two villages at a time (one village from each study arm), and proceed to the next two villages upon completion, and so on. Prior to beginning recruitment in a village, members of our research team will meet with political, religious, and other village leaders, and identify VHT members to will serve as “mobilizers;” together VHTs and community leaders will inform the residents about HBHCT and the upcoming study. Using existing lists of households and maps of homesteads within the village, VHT mobilizers will visit all households in the village and seek permission for the HBHCT team to visit. For the households who agree to have the team visit, the VHT will set an appointment for this visit. The VHT will accompany the team to the household on the scheduled visit day, introduce the team and then leave to allow the recruitment, informed consent, data collection, and HIV testing to occur. The teams will set appointments to re-visit households in which household members were absent. The HBHCT teams consist of a study interviewer, a laboratory technician, and an HIV counselor who were all previously trained in general research procedures. Additionally they were trained in the study protocol and procedures.

#### Eligibility, recruitment, and informed consent procedures

##### HBHCT and brief baseline questionnaire

After the team is introduced by the VHT during the HBHCT visit, the interviewer will verbally screen household members for eligibility for HIV testing and study participation using a brief questionnaire. Eligibility for the baseline interview includes being 18-59 years of age or an emancipated minor, accepting HIV testing, speaking Luganda—the predominant language spoken in the study area, or English and residing in the household. Individuals who don’t normally sleep in the house will be excluded because they may live outside of the study area). We will obtain written informed consent for conducting the baseline interview and HIV testing. Those who consent will be interviewed before being tested for HIV as it would be insensitive to ask participants to answer interview questions after learning they are HIV positive. We expect approximately 25,000 individuals will participate in this aspect of the study.

##### Intervention trial

Individuals who participated in the brief baseline questionnaire interview and are identified as newly diagnosed HIV positive by our HBHCT team, as well as individuals who disclose as being known to be HIV positive during the baseline questionnaire and are confirmed positive in re-testing by the HBHCT team, but have never linked to HIV care, will be eligible for participation in the intervention trial. Only one person per household will be included in the intervention trial to ensure independence in the data. Using computerized random selection, we will randomly select one of the eligible HIV-positive household members for inclusion in the intervention trial. For ethical reasons the same follow-up support (intervention or control) will be offered to other newly diagnosed or HIV-positive household members not in care as will be provided to the household member included in the intervention study. A total of approximately 600 participants (approximately 300 in intervention and 300 in control arm villages) who are eligible and provide written informed consent to participate in the intervention trial will be enrolled.

##### CD4 and HIV VL testing only

We anticipate that approximately 700 participants who complete the baseline questionnaire and test HIV positive will be excluded from the intervention trial because they are already aware of their status and have linked to HIV care, or because there are multiple new HIV diagnoses within a household. These individuals will be offered participation in a “CD4 and VL testing only” group. Including this group will allow us to have HIV VL measures from all consenting new or known PLHIV in the included villages which provides us the ability to assess the population-level effects of the intervention. Study staff will obtain written informed consent for the collection of CD4, VL, and a brief questionnaire interview at 12-month follow-up. See Fig. 1 for a flowchart depicting the study procedures and sample size approximation per study arm.

#### HIV testing procedures

HBHCT will be conducted according to the MOH protocol for HBHCT [41] by study staff trained in HIV testing and counseling procedures. According to the protocol, counseling consists of pre-test counseling and giving results with post-test counseling. Pre-test counseling includes: reviewing the test process, discussing the understanding of potential test results, and assessing risk for HIV infection. Rapid tests will be used which provide results in approximately 15 min. In the measures section we provide details on the HIV testing algorithm. When the results are ready, the counselor will provide the test results to the individual (or couple if applicable). The information provided will depend on whether the test is positive or negative. If the result is negative, the counselor will discuss HIV prevention (condom use, ways to reduce risk) and encourage future testing. If the result is positive the counselor will provide supportive counselling, discuss disclosure (if applicable), emphasize the importance of partner testing (if applicable), discuss HIV care and treatment available at the nearest health facilities and the laboratory technician will take a blood sample for CD4 and HIV VL testing. After the counselor tells the client his/her HIV test results, for those who are newly diagnosed, counselors will provide linkage to care following either the standard-of-care (paper-based referrals) or the PATH/Ekkubo intervention protocol, as detailed in the following sections. Control counselors will be trained to deliver standard-of-care linkage only, and will work only in villages allocated to the control arm. The intervention counselors will be trained in the PATH/Ekkubo intervention protocol over a four-day training and will work only in intervention villages.

### Description of the study arms

#### Control arm: Standard-of-care linkage (see Table 1)

##### During HBHCT

In villages randomized to the standard-of-care arm, the control counselor will provide information on HIV care and treatment and provide the client with a referral to HIV care and a list of health facilities, including location and HIV clinic hours, in their area that provide free HIV care.

**Table 1:** Linkage to care in the control arm: Standard-of-Care

Baseline HBHCT visit 10 min	Provide information on HIV care and treatment available including HIV clinic locations and hours and provide referral to care
Two-Week CD4 results home visit 15 min	Provide CD4 results and a paper-based referral to care. Explain logistics of how to get care and importance of accessing HIV care.

Note: Control arm activities follow standard of care for linkage referrals in Uganda. *HBHCT* home-based HIV counseling and testing

##### Two week CD4 results home visit

Two weeks after HBHCT, the control counselor will return to the participant’s home and provide them with their CD4 test results, and explain that they should take the CD4 results card with them when they present for HIV care. The counselor will also explain what the CD4 count means and if it indicates that they are eligible for ART, until the district adopts the MOH guidelines for immediate ART initiation, at which point they will be told they are eligible for ART regardless of CD4 test result. The two-week interval of return of CD4 results was the standard-of-care before “test and start” began to be adopted. With expansion of universal treatment, CD4 testing will no longer be a requirement for treatment initiation.

#### PATH/Ekkubo intervention arm: Enhanced linkage to care and treatment (see Table 2)

The intervention was originally developed and tested in a randomized trial with urban outpatients receiving opt-out HIV testing by Dr. Wanyenze [39]. Due to logistical differences between outpatient opt-out testing and HBHCT, we have made adjustments to include additional counselling sessions, to encourage and facilitate disclosure and seeking social support to address HIV-related stigma to increase the intervention’s effect on multiple outcomes--beyond those examined in the previous trial. The development of stigma-reduction elements of the intervention were guided by the HIV Stigma Framework [42].

The overall goal of the PATH/Ekkubo intervention is to identify and reduce barriers to HIV care through multiple follow-up counseling sessions following HBHCT. The intervention content includes up to six sessions delivered over a 10-week period. The core components and session schedule are described in Table 2. The first session begins after post-test HIV counseling during the initial HBHCT home visit and subsequent sessions are at 2 weeks and 6 weeks post HBHCT with optional sessions at 8 and 10 weeks for those who have not linked to care by 6 weeks; in addition there is a booster counseling session at the clinic when the client first presents to the HIV clinic. The components of the intervention include: linkage to care and referral, provision of CD4 results during the two-week visit, orientation to the HIV care system and the assessment of potential for adverse effects related to HIV diagnosis and linkage to care (e.g., intimate partner violence). The counselor assesses needs and barriers to linkage (e.g., coping skills, social support, HIV-related stigma, and additional barriers), provides counseling and referrals to overcome identified barriers, and provides additional help planning for disclosure, seeking social support, and dealing with HIV-related stigma. Finally, the session ends with the elicitation of the client’s intention to seek HIV care. Each subsequent session includes reassessment of linkage status and barriers to care and counseling to overcome barriers, building on the counseling provided in the previous sessions. If at any point during the intervention period the client has linked to HIV care, the counselor aims to reinforce engagement in care and assess/reduce barriers to continued engagement.

**Table 2:** Intervention arm: Enhanced Linkage to Care

	Intervention session core components
Session 1, Baseline home visit *Protocol Components 1-8* 30 min	1. Explain linkage to HIV care 2. Assessment of potential adverse effects 3. Assess needs/barriers to HIV care 4. Provide counseling to overcome barriers a. Assess HIV-related stigma b. Counseling to overcome stigma c. Identify sources of social support d. Provide referrals for social support e. Counseling to address other barriers 5. Help the client plan for disclosure, seeking social support, and HIV stigma a. Create a disclosure plan b. Identify treatment supporter(s) c. Encourage support seeking 6. Orient client to the HIV care system and process 7. Elicit client’s intention to seek HIV care 8. Set the time/date for two-week CD4 result home visit
Session 2, Two-week CD4 results home visit 30 min	1. Provide CD4 results and explain what they mean. If not eligible for treatment explain importance of repeat CD4 testing every 3 months 2. Review plans and discuss client’s plans from Session 1 3. Assess if client has linked to care 4. Assess the participant’s needs/barriers to care and provide counseling/referrals a. If patient has not been linked: As appropriate, repeat components 1-7 from Session 1 b. If patient has been linked: As appropriate, repeat components 1-6 from Session 1 in relation to continued linkage 5. Assess HIV-related stigma and if client has disclosed and sought social support. 6. As needed provide counseling on seeking social support 7. Assess and discuss disclosure 8. Discuss treatment supporter 9. Review barriers and plans for overcoming barriers and getting social support 10. Elicit client’s intention to seek HIV care/attend second clinic visit 11. Set the date/time for the next home visit
Session 3, Six-week home visit 30 min	Repeat steps outlined in Session 2
Session 4, First HIV clinic visit 30 min	1. Congratulate client on attending the HIV clinic 2. Assess client’s barriers to attending a second clinic visit 3. Assess if client sought social support and if needed provide counseling on seeking social support 4. Address any other barriers to care and any medical needs 5. Review barriers to care, plans for overcoming barriers and getting social support 6. Elicit intention to attend the next clinic visit
Optional Session 5 & 6, Additional visit/phone calls 30 min	Up to two calls those lost to follow up, repeat Session 3 If cannot be reached via phone, one home visit for those lost to follow up, repeat Session 3

### Data collection procedures and measures

Using a computerized structured questionnaire, a brief baseline interview will be conducted during the HBHCT visit for all participants consenting to HIV testing and interview. Following completion of the interview, the team will undertake HIV counseling and testing following the Ugandan MOH protocol for HBHCT [41]. If the result is positive and the participant has provided informed consent for enrollment in either the intervention trial or the CD4 and VL monitoring only group, the health worker will take blood for CD4 and HIV VL testing. Follow-up interviews will be conducted at six and 12-months follow-up for participants enrolled in the intervention trial, using a computer-based structured questionnaire (<60 min duration). The data collection schedule, procedures, and instruments will be identical in both study arms and follow-up interviewers will be blinded to study arm assignment. A brief, 12-month questionnaire will be administered with participants in the CD4 and VL only group. For both intervention trial and CD4 and VL groups, we and will also take a blood sample for CD4 and HIV VL testing at 12 months, which will be returned to all participants during a 14-month home visit. Additionally, study staff will abstract data regarding attendance at the HIV clinic, CD4 count, and treatment regimen from medical records at each of the participating clinics as a secondary data source for some of the study outcomes. Cost data will be collected from activity logs and accounting records, and through micro-costing of intervention activities. Details of the study variables, measures, and timeframe for collection are described below and in Table 3.

- *HIV status* (baseline). Health workers will obtain a finger stick capillary blood sample to run the HIV rapid tests. Following WHO recommendations to assure HIV testing quality in high prevalence settings [43] and the Ugandan MOH recommended assays, the rapid HIV testing algorithm we will be using in the study is as follows: Determine HIV-1/2 Assay (Alere/ Abbott Laboratories, Chiba, Japan) as the first test in the algorithm. If Determine is non-reactive, an HIV-negative result will be reported. For those found reactive with Determine, a second test, HIV 1/2 STAT-PAK (Chembio Diagnostic Systems, Medford, NY, USA) will be performed. If reactive with STAT-PAK, results will be reported as HIV positive. If reactive with Determine and non-reactive with STAT-PAK, both the Determine and STAT-PAK tests will be repeated. If both the repeat Determine and repeat STAT-PAK are reactive, an HIV-positive result will be reported. If both the repeat Determine and repeat STAT-PAK are non-reactive, an HIV-negative result will be reported. If the repeat Determine is reactive and the repeat STAT-PAK result is non-reactive, the sample will be tested using Uni-Gold HIV (Trinity Biotech, Bray, Ireland). If Uni-Gold is reactive, an inconclusive result will be reported and the participant will be re-tested in 14 days. If Uni-Gold is non-reactive, an HIV negative result will be reported. For quality control purposes, a venous blood sample will be taken with every 10th participant who tests HIV negative. Deoxyribonucleic acid polymerase chain reaction tests will be performed on these specimens for quality control.

#### Primary outcome measures

- *Individual and population HIV VL suppression* (baseline and 12 month). We will examine HIV VL suppression at two levels; VL <200 and as undetectable VL —defined as HIV RNA <20 copies/mL. We will also examine changes in VL between baseline and follow-up to determine reductions considered statistically significant—a threefold, or a 0.5 log_10_ copies/mL reduction [44]. Specimens for VL testing will be collected via venous blood draw at baseline and during the 12-month in-home follow-up interview. HIV VL testing will be performed on plasma processed at the Gombe Hospital Laboratory and subsequently sent to Uganda Virus Research Institute (UVRI). HIV VL testing at UVRI will be done using the Roche COBAS AmpliPrep/TaqMan assay (Roche Molecular Systems, Pleasanton, CA) which has a lower limit of detection of 20 copies/mL.

#### Secondary outcome measures

Our main secondary outcome measures include HIV care linkage, ART initiation, and short-term retention in care (detailed below) collected through participant self-report at six and 12-month follow-up interviews, supplemented by clinic records and direct observation of participants’ HIV clinic cards and ART pill bottles where possible.

- *Linkage and time to care* (six and 12 months, clinic records). We will assess linkage to care with data on: enrollment in an HIV clinic, having a second HIV clinic visit, and time from HIV testing to enrollment in care at an HIV clinic.
- *ART initiation & time to initiation* (six and 12 months, clinic records). To assess receipt of HIV treatment we will determine if participants have been prescribed and have taken ART and the time from HIV testing to ART initiation. We will determine the percentage of participants eligible for ART (currently CD4 < 500 until the guidelines [8] to expand eligibility are implemented nationwide), who received antiretrovirals (ARVs) based on their CD4 count taken at the time of HBHCT, as well as the time from diagnosis to ART initiation. If possible, interviewers will directly observe the participant having ARVs at their home and review the patient’s HIV clinic record card to confirm self-reported data.
- *Short-term retention in care* (six and 12 months, clinic records)*.* We will assess the following retention measures that have demonstrated the best discriminatory capacities for predicting viral suppression [45, 46]: a) missed visit count: number of missed visits accrued (count measure) based on scheduled visits determined by MOH clinical guidelines, b) visit adherence: proportion of kept visits/scheduled visits (kept + missed visits) (continuous measure, range = 0.0–1.0), and c) four-month visit constancy: number of four-month intervals with at least one kept visit (categorical measure, range = 0–3).

#### Independent variables

- *Sociodemographics* (baseline). We will assess participants’ gender, age, tribe, educational level, socioeconomic status, employment status, income, religion, gender, marital/partnership status, distance to clinic, and travel time to the clinic.
- *Prior use of health services* (baseline). We will measure prior use of HIV services, including history of HIV testing, frequency, location, and reasons for health service use in the prior 6 months, male circumcision for men, and location of births for women.
- *CD4 cell count* (baseline, 12 months)*.* CD4 cell count will be assessed via venous blood draws along with VL at baseline and 12-month follow-up. Baseline CD4 values may affect viral suppression success [47]. We did not include CD4 as an outcome measure because according to recommendations by WHO and the United States Department of Health and Human Services, when VL is available, CD4 has little added value for monitoring treatment adherence and efficacy of ART [44, 48].
- *Tuberculosis (TB)* (six and 12 months, clinic records). We will assess co-infection with TB using questions adapted from the MOH’s HIV clinic card. Interviewers will use the participant’s clinic card and self-report to record if the participant is TB infected, date of diagnosis, and treatment initiation and regimen.
- *ART adherence* (six and 12 months). We will assess self-reported adherence to ART using the Adult AIDS Clinical Trials Group scale [49], which includes recall questions about ARVs missed for the previous 4 days prior to the interview, as well as 17 items assessing participants’ main barriers to adherence. It has demonstrated construct validity in Uganda and similar settings [50].
- *Disclosure of HIV-test results* (six and 12 months). We will measure HIV status disclosure with an instrument used in prior research in Uganda and other African countries [39, 51]. It asks about the disclosure of serostatus to spouses, sexual partners, family members, community members/leaders, physicians, etc. The proportion of participants reporting each disclosure is calculated after eliminating those who say the disclosure was not applicable to them.
- *Receipt of instrumental and emotional social support* (six and 12 months). We will measure receipt of instrumental and emotional social support using the Social Support Scale [51], which contains six items on emotional support and four items on instrumental social support. This scale was adapted from the Duke-University of North Carolina Functional Social Support Questionnaire [52] and has been validated among PLHIV in rural Rwanda (α = 0.91) [53]. In addition, we will determine if participants have a treatment supporter, have attended HIV support groups, or been counseled by an HIV-positive peer educator, and how helpful each of these forms of counseling have been on a four point scale ranging from “not helpful” to “very helpful.”
- *HIV stigma* (baseline, six, and 12 months). At baseline, for those individuals reporting they are HIV negative or do not know their HIV status, we will measure enacted stigma, including nine items on discrimination, stereotypes, and prejudice using Kalichman et al.’s [54] AIDS-related stigma scale, which has been validated in South Africa [54]. We will also measure anticipated stigma, asking participants how they would feel if they tested positive, using six items from Earnshaw’s [42] anticipated stigma scale and six items from Berger et al.’s [55] HIV stigma scale. We will calculate the mean of all participants from each village to get a village (cluster)-level HIV stigma score at baseline. At six and 12-month follow up, and at baseline for those who report already knowing they are HIV positive, we will assess the following dimensions of HIV stigma among PLHIV [56]: (a) anticipated stigma using six items from Earnshaw’s [42] anticipated stigma scale and two subscales from Berger et al.’s [55] HIV stigma scale: eight items assessing anticipated stigma related to disclosure and eight items assessing anticipated stigma related to concern with public attitudes about PLHIV; (b) enacted stigma using six items from Earnshaw’s scale [42], 16 items from the personalized HIV stigma subscale of Berger et al.’s [55] scale, as well as items from Sayles et al.’s [57] multidimensional HIV stigma scale: 12 items from the stereotypes subscale and seven items from the social relationships subscale; and (c) internalized stigma using Kalichman et al.’s [58] internalized AIDS-related stigma scale. All of these measures have shown good reliability among sub-Saharan African samples (α = 0.73-0.78) [59–61].
- *Subjective health status* (six and 12 months). We will use the 36-item Medical Outcomes Study-HIV short form [62] to assess subjective health status adapted for the Ugandan context [63] and shown to be reliable among PLHIV in rural populations in Uganda (α > 0.70) [63, 64].
- *Perceived need for HIV care/treatment* (six and 12 months). We will adapt five items used in a study in Zambia [65] to assess participants’ perceived need for HIV treatment. The items assess participants’ agreement with statements such as: “You do not need to go to the HIV clinic because you feel healthy” and “You do not want to take any medicine.”
- *Barriers to care* (six and 12 months). We will measure barriers to attending HIV care, including distance/time to the clinic, financial barriers, work/child care responsibilities, wait times, and concerns with privacy among health workers. We will include 18 items adapted from the LifeWindows Information Motivation Behavioral Skills ART Adherence Questionnaire [66] and Smith et al.’s [67] scale on engagement and retention in pre-ART HIV care. We will also determine the actual distance and travel time from the participants’ home to the HIV clinic and for the cost of transportation to the clinic, for those who are able to pay.
- *HIV care system literacy.* We will use nine items from the Brief Estimate of Health Knowledge and Action HIV Version scale [68] to assess participants’ knowledge of the HIV care system, including questions on where they can get treatment, the cost of treatment, treatment eligibility, etc.
- *Beliefs about traditional and western medicine* (six and 12 months). We will assess beliefs towards western and traditional medicine and use of traditional medicine to treat HIV using items adapted from the treatment denialism subscale of Kalichman’s AIDS denialism scale [69].
- *Relationship with the clinic provider*. We will measure participants’ satisfaction with their healthcare provider using 10 items from a scale used in South Africa by Westaway and colleagues [70].
- *Acceptance of HIV diagnosis.* Three items modified from the Brief COPE Scale [71] will be used to assess participant’s acceptance of their HIV diagnosis by asking participants if they have refused to believe their test results since they tested positive.
- *Alcohol use*. We will assess alcohol use using the Alcohol Use Disorders Identification Test [72], shown reliable among PLHIV in rural Uganda (α = 0.71) [73].
- *Depression* (six and 12 months)*.* We will measure depression symptoms using a modified 20-item version of the Center for Epidemiological Studies scale [74], which has been shown to be reliable in a rural Ugandan sample (α = 0.90) [75].
- *Positive and negative life events following HBHCT* (six and 12 months). We will include items to assess life events following HIV testing using items by Grinstead et al. [76] and used in our prior work in Uganda [39]. The scale assesses the occurrence of life events since diagnosis, such as the strengthening of a relationship, breakup of a relationship, physical abuse by a sexual partner, and neglect by family.
- *History of intimate partner violence* (six and 12 months). Items assessing experience of intimate partner violence originating from the Conflict Tactics Scale [77] and included in the WHO multicountry study [78] will be included. The scale includes 10 questions asking participants if they experienced any emotional (e.g., has he/she ever insulted you or made you feel bad about yourself?), physical (e.g., has he/she ever kicked you, dragged you, or beaten you up), and sexual violence (e.g., has he/she physically forced you to have sexual intercourse when you did not want to?) from a partner ever, and in the prior 12 months.
- *Intervention contamination* (six and 12 months)*.* We will ask participants what type of counseling they received and from whom (to assess potential contamination) and the number of HIV-positive household members and if they are in care and on treatment. We will keep records on the number of and duration of intervention sessions each intervention arm participant actually received.
- *Clinic level factors* (twice monthly)*.* We will monitor ART and cotrimoxazole stock outs at the participating clinics and hospital, where participants will most frequently visit to pick up their medications, on a twice-monthly basis through use of a checklist that includes of all drugs routinely prescribed for HIV-positive individuals.

#### Cost measures

We will collect cost data to determine total intervention cost under enhanced linkage and standard-of-care arms, as well as the costs of HIV-related health services or other costs incurred up to 12 months following the receipt of HBHCT, using methods previously developed by the research team [9, 79]. Costs will be assessed in three categories: (i) direct costs of HBHCT & linkage activities, (ii) HIV care costs incurred as a consequence of HBHCT & linkage activities, and (iii) time-costs and out-of-pocket expenses incurred by individuals receiving the intervention. For the direct costs of HBHCT and linkage activities, data on personnel time and consumables will be extracted from staff activity logs, and data on training costs, transportation, and management/administrative overheads will be collected from program records. For the costs of additional HIV care and laboratory services incurred as a consequence of the intervention, costing will be undertaken at HIV clinics to establish unit costs for clinic visits, ART and other medicines, and routine laboratory tests. The number and timing of HIV-related services received by study participants will be collected from clinic records. Wages and other input prices will be based on public sector values to ensure that results are applicable to routine program settings. During the 12-month follow-up interviews we will include questions to determine costs incurred by study participants in relation to receipt of the intervention and HIV care (including time costs, travel costs, and any out-of-pocket payments related to HIV care).

#### Monitoring intervention fidelity

Counselors will use electronic intervention protocols programmed on tablets to guide them in completing the components of each intervention session. For each step they will document its completion by selecting potential outcomes or typing in a description, e.g., “primary barrier is fear of being seen at clinic;” “no noted potential adverse events.” This will allow us to capture the percentage of intervention protocol steps that the counselor delivered. We have used this method to monitor intervention fidelity in previous intervention research in Uganda [80].

**Table 3:** Data collection, measures, data sources

Data element	Timeframe	Data source/ instrument	Data collected	Collected from
Brief demographic assessment	Baseline	Self-report	Sociodemographics (gender, age, education, economic and marital status, religion, etc.); prior access to health services (e.g., HIV testing, circumcision among men); HIV stigma; fertility desires and contraception (pregnancy status, number of living children, number of additional children wanted, contraceptive use)	• All individuals consenting to HBHCT and baseline questionnaire interview
Follow-up assessments	6 months, 12 months	Self-report Clinic records^a^	Linkage and time to HIV care^a^; co-trimoxazole and ART initiation & time to initiation^a^; short-term retention in care^a^; tuberculosis^a^; ARV adherence^a^; HIV disclosure; receipt of instrumental and emotional social support; HIV stigma (anticipated, enacted, internalized; subjective health status; perceived need for treatment; barriers to accessing care; health care system literacy; beliefs about traditional and western medicine; patient-provider relationship and clinic wait time; acceptance of HIV diagnosis; alcohol use; depression; positive and negative life events following HIV testing; intimate partner violence	• Intervention trial participants (intervention and control group)
Brief follow-up assessment	12 months	Self-report	Clinic attendance; CD4 results, taking co-trimoxazole and ART	• Viral load and CD4 only group
Biological measures	Baseline, 12 months	Laboratory report	• HIV status (baseline) • Viral load (baseline, 12 months) • CD4 cell count (baseline,12 months)	• Intervention trial participants (intervention and control group) • VL and CD4 only group
Clinic-level data	Twice monthly	Checklist	Drug stock-out: Availability of all ARVs and cotrimoxazole	• All participating HIV clinics
Costing data – service utilization	Ongoing (collected in program records and clinical data)	Program records Clinic records Log-book	• Receipt of HBHCT home visits, counseling visits, follow-up contacts • HIV care services (clinic visits, lab tests, ARVs)	• Intervention trial participants (intervention and control group)
Costing data – service unit costs	Year three of trial	Program accounts, micro-costing at HIV clinics	Intervention related costs – personnel time, consumables, infrastructure, overheads	• Study administration, participant HIV clinics
Costing data – patient costs	12 months	Self-report	Costs incurred by participants related to intervention and/or HIV care	• Intervention trial participants (intervention and control group)
Intervention fidelity	Daily	Electronic protocol checklist	Percentage of intervention sessions steps completed	• Intervention counselors

Note: ^a^ indicates follow-up assessment items measured through both participant self-report and clinic records. ARV: antiretroviral; *HBHCT* Home-based HIV counseling and testing; VL: viral load

### Data analysis approach

Univariate statistics will be conducted on primary and secondary endpoints as well as covariates to describe the sample characteristics for the two study arms. Attrition in the two arms will be compared. Multiple imputation technique will be implemented and sensitivity analysis will be conducted.

To assess the primary outcome (the proportion of participants who are virally suppressed at 12-months) and secondary outcomes (linkage to care, time to care, receipt of opportunistic infection prophylaxis, and initiation of ART among those eligible for ART at baseline), linear mixed models and nonlinear mixed models will be applied. Information criterion such as Akaike information criterion or Bayesian information criterion will be used for model selection.

In evaluating the intervention effectiveness mechanisms, and mediators and moderators, we will use structural equation modeling to estimate the causal effects using growth mixture modeling to account for the longitudinal and clustered data. To assess an existence of moderators of an intervention effect, covariates such as disclosure, social support, HIV stigma, adherence, distance from the clinic, CD4 count, having other HIV-positive household members, number and duration of intervention sessions received, village-level stigma towards PLHIV, etc. will be included. A nonlinear mixed model will be fitted independently for each covariate plus the treatment variable and an interaction term between the covariate and the treatment.

To examine the natural history of stages in the HIV care continuum, we will use the control group to identify barriers to and facilitators of linkage to care and treatment, retention, and viral suppression, potential predictors include: sociodemographics, HIV stigma, disclosure, etc. Stepwise forward selection method from both logistic regression model and general linear model will be used to identify potential significant predictors for our analyses. Predictors that are selected from the stepwise method will be included in our mixed models.

### Sample size justification and power analysis

The sampling scheme is stratified clustering with matched-pair sampling. Within each area, we pair matched clusters (villages) by distance to the HIV clinic and randomly assigned one village to be in the intervention arm. We have ensured that villages assigned to different study conditions are not within 4 km (1 h by foot) of each other to avoid possible discussions between different arms of participants.

We have powered the study to detect differences between the intervention and the standard-of-care for the primary outcome of HIV viral suppression (< 20 copies/ml) as well as intermediate outcomes: linkage to care and time to care, and, among those eligible for ART: time to and receipt of ART. Using census data, HBHCT acceptance rates from our own and other prior studies [22], HIV prevalence and prior knowledge of HIV status in this area from the recent prevalence survey [17], and estimating a 92% acceptance rate for enrollment of eligible participants into the study we calculated that the average cluster size (number of newly-diagnosed PLHIV per village who enroll in the study) will be 14 (range 8 to 37). In calculating the necessary clusters, type I error rate, desired power of tests, measure of effectiveness between the intervention and control group, cluster size, and coefficient of variation (k) or intraclass correlation coefficient are needed. We estimated viral suppression at 12-month follow-up among those eligible for ART at baseline to be 55% in the standard-of-care arm and 75% in the intervention arm. This estimate takes into account 12-month viral suppression success rates from SSA public sector ART programs [45, 81–84] and our own [23, 39] and others’ data on intermediate outcomes of linkage to care and ART initiation [22, 28]. With a type I error rate of 0.05 (two sided), average cluster size of 14, power of 80%, 12% difference in outcomes, and coefficient of variation of 0.24, we need 40 clusters after accounting for a 18% attrition rate for the follow-up. We expect that the 40 clusters will yield approximately 600 PLHIV participants across the two study arms.

### Ethics

Approval of all study procedures has been obtained from the institutional review boards at San Diego State University and Makerere School of Public Health. Study approval has also been received from the Uganda National Council for Science and Technology. We will obtain written informed consent for participation in HIV testing and the brief baseline questionnaire interview. For individuals who want HIV testing, but do not want to participate in the brief questionnaire interview, verbal informed consent with a waiver for written consent will be obtained. Separate consent forms will be used to obtain written informed consent for all participants enrolled in the intervention trial or CD4 and VL Only arm.

We expect that the overall risk:benefit ratio will be favorable to patients participating in the study, and the risks related to HBHCT are not greater than the risks associated with the standard-of-care health system procedures related to home-based health services, which is commonplace in this rural Ugandan setting. Among the main risks of the study, are minimal physical risks associated with fingerstick and venous blood draws (bruising, bleeding, discomfort, lightheadedness, and potential infection), which are not greater than the risks incurred from normal medical blood testing procedures. We will minimize these risks by hiring qualified staff who also have experience in phlebotomy and finger stick blood collection to perform these procedures.

There is also the potential for breaches in confidentiality related to collected data (detailed contact information of patients, interviewer-administered computerized questionnaires, data extracted from medical records, HIV results, CD4 and VL test results). To reduce the potential for recorded data to identify the participant, we will (a) use unique identifiers (random 6 digit study identification (ID) numbers) instead of medical ID/record numbers or participant names to identify all data; (b) store of the list that links the participants’ names to their unique identifiers in a locked, secure location; (c) store participant contact details in a locked cabinet separate from the list that links participant names to study ID numbers and it will not contain the unique study ID number, (d) use encryption protocols and password protection for all data collected and/or stored electronically and encryption procedures to securely transfer data, and (e) train all study staff in the importance of and procedures for protecting participants’ confidentiality.

Participants in our study may experience discomfort while discussing sensitive information during interviewer-administered computerized questionnaires (e.g., domestic violence) and intervention counseling sessions (e.g., barriers to attending HIV care). These risks will be mitigated through clearly informing patients of their right to refuse to answer any question or engage in any discussion that makes them uncomfortable and providing adequate training to counselors and interviewers in navigating sensitive discussions. For participants receiving the intervention, the possibility of experiencing an unforeseen unintended negative consequence from participating in the intervention (e.g., negative reactions to their disclosure of HIV status). This risk will be mitigated by training counselors delivering the intervention to conduct counseling sessions only in private settings and to use judgement in which strategies are appropriate for participants based on their personal circumstances. Finally, there is potential for public disclosure of the HIV test result. However, HIV test results, like all other data, will only be identified by a randomly generated study ID number. We discuss ethical concerns and protections for participant confidentiality further in the discussion section.

## Discussion

We hypothesize that compared to individuals receiving standard-of-care, or paper-based referrals, individuals receiving the PATH/Ekkubo intervention will be more likely to be virally suppressed 12 months after HBHCT, and demonstrate shorter time to HIV care and ART initiation among those eligible. Individuals receiving the PATH intervention will receive multiple follow-up counseling sessions aimed to address barriers to HIV care. This study will indicate whether enhanced linkage to care counseling found effective in improving linkage to HIV care during facility-based HIV testing in an urban population [39] can similarly improve linkage to HIV care, and subsequently reduce VL, when integrated into HBHCT in a rural setting. This study will also provide further information on the cost-effectiveness of this approach compared to providing standard-of-care paper-based referrals during HBHCT.

The evaluation of this intervention has the ability to advance science and improve public health service delivery in several ways. To date, few randomized controlled trials have assessed interventions to improve linkage to care in the context of HBHCT, leaving a gap in our understanding of the best approaches to promote linkage to HIV care and treatment in HBHCT in SSA countries. For those in the intervention arm, it is anticipated that this study will help patients reduce barriers to accessing HIV care by providing strategies and resources to overcome stigma and other barriers, as well as the requisite knowledge, motivation, and self-efficacy needed to do so. If found effective, our study will provide information on the mechanisms through which our intervention influenced linkage behavior, which will be useful in the development of future interventions. Specifically, we will test the hypothesis based on prior research, that social support will have a protective effect against enacted and internalized stigma [32, 33, 38] and additionally test the subsequent effects on linkage to care, receipt of HIV treatment, and viral suppression. In addition, by treating the control-arm as a natural history control, our study will further contribute to the scientific literature on barriers to and facilitators of linkage to care and treatment and HIV viral suppression. This data, together with our findings on costing, have the potential to inform the integration of cost-effective avenues for follow-up and the delivery of support for HBHCT in Uganda and similar settings.

### Confidentiality and ethical considerations

Conducting a CRCT in the setting of HBHCT has unique ethical challenges surrounding confidentiality of participants’ HIV status. Given the nature of our intervention, which includes multiple home visits for PLHIV, efforts to minimize the risk of participants being identified as HIV positive in their community or families through participation in our study are paramount. In addition to commonly employed safeguards to protect participant confidentiality, such as the use of unique identifiers linked to personally identifying information and the careful storage and transfer of sensitive data (as described previously), we describe here issues of confidentiality and ethics specific to conducting research with PLHIV during HBHCT, which may also be relevant to other community-based trials that involve PLHIV, and how we’ve aimed to address them.

With research involving PLHIV, there is always small risk of public disclosure. In our study, home visits from counselors could increase risk of public disclosure. While we think it is unlikely that visits from counselors in it of itself would signal one’s HIV-positive status to others in the community, as visits from counselors and various health workers are common in Uganda for a variety of healthcare reasons (e.g., family planning, postnatal care, malaria care, immunizations), we will minimize this risk in several ways. Firstly, when counselors do home visits to HIV-positive participants for the intervention trial, they will also visit other homes nearby.

Secondly, only those who have tested HIV positive and are enrolled in the intervention trial will know that the intervention trial is about linkage to HIV care. Participants in the baseline interview and HIV testing and those who only want HIV testing will not be informed about the intervention trial and thus will not know that those who are positive and in the intervention trial will be getting home visits. The baseline assessment itself will be framed more broadly as a survey on health care access and will include questions on other health service uptake beyond HIV (e.g., family planning) to mask the study’s purpose. Masking the purpose of the study requires that we consent participants for the intervention trial after they have learned they are HIV positive.

In addition to potential breaches in confidentiality among community members, counselor visits also have the risk of family members or others overhearing discussions in which sensitive information is being addressed. Training counselors to ensure privacy during home visits, as well as training counselors to use their judgment in rescheduling a home visit if privacy is compromised will minimize this risk.

Although this risk is considered to be low, some strategies that patients elect to implement in order to engage in HIV care may potentially have unintended negative consequences on the study participant or others. To minimize this risk, counselors will be trained to respect patients’ decisions and their comfort levels with HIV care linkage strategies and goals, emphasizing the decision to engage in HIV care, disclose one’s HIV status to others, and seek social support will be a personal one of the patient. When assessing linkage behavior, all components of a patient’s physical health, mental health, and social situation will be considered when discussing linkage strategies, including the threat of domestic violence. A variety HIV care linkage strategies will be discussed with each participant to minimize the possibility that a particular plan to access care may place a patient in any danger. Participants will choose the one least likely to cause negative consequences. Counselors will screen for the threat of domestic violence when deciding on a disclosure plan and linkage strategies during the first counseling session, and will conduct a brief risk assessment for domestic violence at every subsequent meeting with participants. Should participants require additional support, counselors will refer participants to relevant staff at the clinic or hospital, who will be equipped to provide appropriate referrals as necessary to study participants for additional counseling, substance abuse issues, domestic abuse, and the threat of violence when these issues arise. Participants who disclose potential abuse (both men and women) will be immediately referred to community and/or onsite services equipped to manage situations of domestic abuse when available or to higher levels when unavailable. Immediate access to crisis intervention and emergency health services, including referrals for safe shelter, are available through the hospital as needed.

### Limitations

Among the study’s limitations is the threat for contamination between intervention and control villages. However, randomizing at the level of the village and having villages assigned to different study arms not geographically close to one another should minimize this threat. We will monitor potential contamination by asking all study participants what kind of linkage counseling they received and from whom. Interviewers conducting the follow-up assessments will be blinded to study condition, however, un-blinding is possible if participants discuss the intervention with the interviewer. We will monitor this by debriefing interviewers. In addition to individual-level data, we will collect clinic-level data; cross-classification may occur if participants seek HIV care at a clinic other than the one nearest to them. However, our data analysis approach can accommodate cross-classification. Finally, in order for participants to be eligible for participation, they must be willing to accept home visits, which may limit the generalizability of the findings. However, we judge this to be only slightly more than in any study which tracks participants via home visits, and from prior studies, we have found that very few patients decline to participate due to the possibility of being visited at home.

## Conclusion

If the PATH/Ekkubo intervention is found effective, the anticipated public health benefits include improving individual health by reducing the time in which participants are linked to care. Timely linkage to care improves treatment outcomes and reduces mortality rates [85–87]. Participation in our study in itself regardless of study condition provides individuals the opportunity to learn their HIV status, and receive diagnosis and treatment for HIV that would have otherwise gone unnoticed and untreated. Moreover, through ART’s effect on VL, increasing the number of PLHIV who are in HIV care and receiving ART will have a secondary outcome of the prevention of ongoing HIV transmission to others [48]. Our study will further add to the scientific literature on the potential population-level health benefits of behavioral interventions, by assessing population-level VL as a study outcome.

## Abbreviations

AACTG: Adult AIDS Clinical Trials Group
AIDS: Acquired Immunodeficiency Syndrome
ART: Antiretroviral therapy
ARVs: Antiretroviral medications
AUDIT: Alcohol use disorders identification test
CRCT: Cluster randomized controlled trial
HBHCT: Home-based HIV counseling and testing
HIV: Human immunodeficiency virus
ID: Identification
MOH: Ministry of health
PATH Study: Providing access to HIV care study
PLHIV: People living with HIV
SSA: Sub-Saharan Africa
TB: Tuberculosis
UVRI: Uganda Virus Research Institute
VHT: Village health team
VL: Viral load
WHO: World Health Organization
